# The Potential Mechanisms of Berberine in the Treatment of Nonalcoholic Fatty Liver Disease

**DOI:** 10.3390/molecules21101336

**Published:** 2016-10-14

**Authors:** Xiaopeng Zhu, Hua Bian, Xin Gao

**Affiliations:** 1 Department of Endocrinology and Metabolism, Zhongshan Hospital, Fudan University, Shanghai 200032, China; zhuxp200@foxmail.com (X.Z.); zhongshan_endo@126.com (X.G.); 2 Institute of Metabolic Disease, Fudan University, Shanghai 200032, China

**Keywords:** berberine, nonalcoholic fatty liver disease, mechanism, insulin resistance, adenosine monophosphate-activated protein kinase

## Abstract

Nonalcoholic fatty liver disease (NAFLD) is a globally observed metabolic disease with high prevalence both in adults and children. However, there is no efficient medication available yet. Increased evidence indicates that berberine (BBR), a natural plant product, has beneficial effects on NAFLD, though the mechanisms are not completely known. In this review, we briefly summarize the pathogenesis of NAFLD and factors that influence the progression of NAFLD, and focus on the potential mechanisms of BBR in the treatment of NAFLD. Increase of insulin sensitivity, regulation of adenosine monophosphate-activated protein kinase (AMPK) pathway, improvement of mitochondrial function, alleviation of oxidative stress, LDLR mRNA stabilization, and regulation of gut microenvironment are the major targets of BBR in the treatment of NAFLD. Additionally, reduction of proprotein convertase subtilisin/kexin 9 (PCSK9) expression and DNA methylation are also involved in pharmacological mechanisms of berberine in the treatment of NAFLD. The immunologic mechanism of BBR in the treatment of NAFLD, development of berberine derivative, drug combinations, delivery routes, and drug dose can be considered in the future research.

## 1. Introduction

Nonalcoholic fatty liver disease (NAFLD), considered as the hepatic manifestation of the metabolic syndrome, is characterized by fat deposition in the cytoplasm of hepatocytes in the absence of overt alcohol consumption or other significant factors of liver injury. NAFLD is a globally metabolic disease with a wide spectrum of pathology ranging from simple steatosis to nonalcoholic steatohepatitis (NASH) and cirrhosis [1]. NAFLD affects 30% of the general adult population and up to 60%–70% of diabetic and obese patients [2,3,4]. Recent studies showed that NAFLD is becoming a major cause of hepatocellular carcinoma (HCC) in the United States [5], and some of HCC cases can occur in the absence of cirrhosis [6,7]. A more recent study showed that the annual incidence of NAFLD between 2003 and 2011 ranged from 2% to 3% in a United States cohort of veterans, and the prevalence of NAFLD changed from 6.3% in 2003 to 17.6% in 2011 [8]. NAFLD is also closely associated with insulin resistance, metabolic syndrome, and type 2 diabetes. The relationship between NAFLD and cardiovascular disease among the general population has been increasingly confirmed [9,10]. Additionally, recent evidence elucidates that patients with NASH are more sensitive to developing colorectal adenomas and advanced neoplasms [11].

So it is necessary to develop an efficient therapy for fighting against or preventing NAFLD. Although some antioxidants, insulin sensitizers, and lipid-lowering drugs—such as vitamin E, metformin, thiazolidinediones, and statins—have been investigated and show a certain effect on reducing liver fat content and liver enzymes, improving partial liver histological lesions and lowering the score of NAFLD, they have no significance in alleviating liver fibrosis [12,13]. Therefore, there is still no approved pharmacological therapy for NAFLD available at present [14]. Recently, the effect of obeticholic acid, a farnesoid X nuclear receptor ligand, has been conducted in adult patients with non-alcoholic steatohepatitis [15]. The result showed that oral administration of obeticholic acid alleviated the liver histology of non-alcoholic steatohepatitis, suggesting the potential efficacy of obeticholic acid in the treatment of NAFLD [15]. However, the benefits and safety of obeticholic acid in the long term are still unknown, which should be elucidated in the future.

Berberine (BBR, C_20_H_18_NO_4_), an isoquinoline quaternary alkaloid, has been widely used in Ayurvedic and Chinese Medicine for hundreds of years [16], which is extracted from *Coptis chiensis, Rhizoma coptidis, Hydrastis canadensis*, etc. [17]. BBR is a natural plant product which is commonly used to treat diarrhea and gut infections in clinical practice. Additionally, a wide spectrum of additional pharmacological effects of BBR—containing the treatment of central nervous system disorders, diabetes, cancer, cardiovascular disease, depression, hypertension, hypercholesterolemia, etc.—have been discovered [10,18,19]. Among them, a number of effects of BBR like regulation of metabolism disorder, increase of insulin sensitivity, lipid-lowering effect, and improving glycometabolism, which may partially indicate its potential role in treatment of NAFLD, attract us to pursue it as a novel natural drug for NAFLD. However, the mechanism of BBR in the treatment of NAFLD has not yet been conclusively determined. With much excellent work done towards this end, great progress has been made in the mechanism of BBR in the treatment of NAFLD, which significantly increases our comprehension of BBR.

In this review, we briefly summarize the pathogenesis of NAFLD and the targets of BBR in the treatment of NAFLD, and focus on the potential mechanisms of BBR in the treatment of NAFLD under the categories of insulin resistance, adenosine monophosphate-activated protein kinase (AMPK) pathway, mitochondrion dysfunction, serum cholesterol-lowering effect, gut microenvironment, and other mechanisms.

## 2. The Pathogenesis of NAFLD and the Targets of BBR in the Treatment of NAFLD

**Figure 1:**
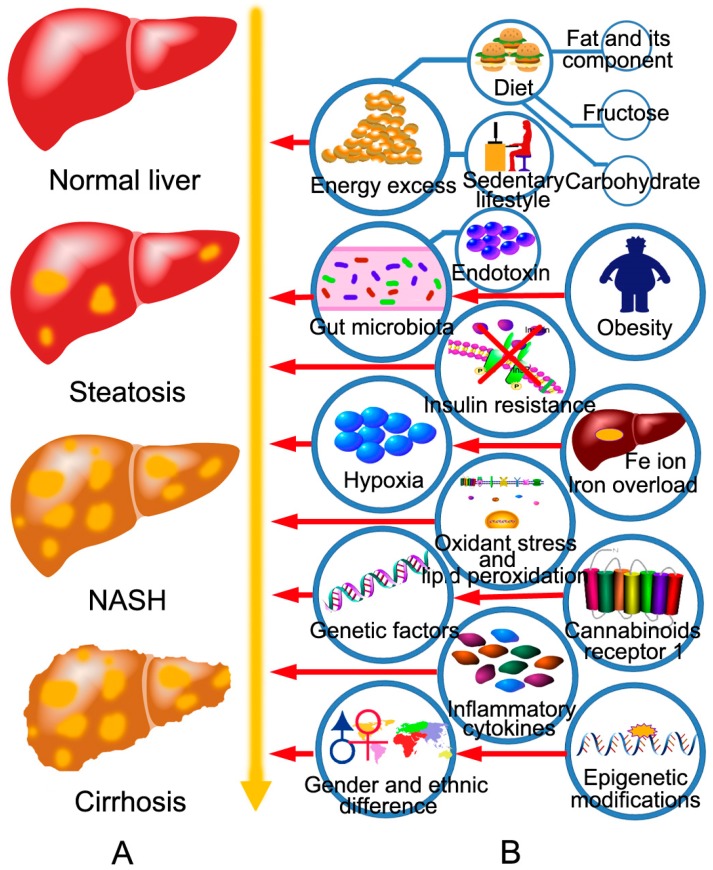
The disease spectrum of NAFLD and the factors that influence the progression of NAFLD. (**A**) The spectrum of NAFLD ranges from simple steatosis to NASH and cirrhosis. Simple steatosis is characterized by the ectopic accumulation of lipid droplets in the cytoplasm of hepatocytes without hepatocyte injury, inflammation, and fibrosis histologically. Steatosis is self-limited and can be reversed by lifestyle modifications. Steatosis can develop to NASH, which differs from simple steatosis in the presence of hepatocyte injury, inflammation, and fibrosis. Long-term NASH leads to cirrhosis. In NASH-related cirrhosis, normal liver structure is damaged and replaced by type 1 collagen, and pseudolobules are formed; (**B**) The factors that influence the progression of NAFLD include energy excess, obesity, insulin resistance, genetic factors, gender and ethnic difference, gut microbiota, hypoxia, oxidative stress and lipid peroxidation, inflammatory cytokines, liver iron overload, endogenous cannabinoids receptor 1, and epigenetic modifications. Energy excess includes diet and sedentary lifestyle.

NAFLD initiates with aberrant accumulation of triacylglycerol (TG) in hepatocytes. Both increased input or synthesis of TGs and decreased output of TGs promote the progression of NAFLD. Increased input or synthesis of TGs includes increased dietary intake, increased lipolysis from adipocytes, and increased de novo lipogenesis in hepatocytes, which can be induced by hyperinsulinemia, hyperglycemia, obesity, and fructose ingestion [20,21,22]. Decreased output of TGs includes reduced β-oxidation in mitochondria, reduced extra-hepatic transport of TG in the form of very low-density lipoprotein (VLDL) [20]. Then, TGs accumulated in hepatocytes induce simple steatosis. Long-term hepatic steatosis can develop to NASH, the mechanisms of which include oxidative stress, maladjustment of the unfolded protein response, endoplasmic reticulum (ER) stress, lipotoxicity, and dysbiosis in gut [23,24,25,26]. Long-term hepatocyte death of NASH contributes to fibrosis. NASH-related fibrosis can lead to cirrhosis, and cirrhosis can develop into hepatic carcinoma (Figure 1A). Additionally, other factors also influence the progression of NAFLD, which have been briefly summarized in Figure 1B.

## 3. The Potential Mechanisms of Berberine in the Treatment of NAFLD

Both animal experiments and clinical trials suggest the potential benefit effect of BBR on NAFLD. BBR significantly decreases serum lipid levels both in rats and patients and induces a mild weight loss in obese patients [27]. Additionally, BBR slightly reverts lipid profile in Caucasian subjects with low cardiovascular disease risk [28]. We carried out a randomized, parallel controlled, open-label clinical trial (NCT00633282) including 184 patients to estimate the efficacy of BBR in patients with NAFLD and found that treatment with BBR plus lifestyle intervention greatly reduced hepatic fat content and improved body weight, homeostatic model assessment for insulin resistance (HOMA-IR), and serum lipid profiles, compared with lifestyle intervention alone [29]. Nevertheless, the precise mechanism underlying these effects is not yet completely uncovered [30].

Generally, both increased synthesis and decreased output of TGs are the targets of BBR in the treatment of NAFLD. BBR can suppress de novo lipogenesis, increase fatty acid β-oxidation, and increase extra-hepatic transport of TG in the form of VLDL in liver. Additionally, liver inflammation and other pathophysiological processes such as oxidative stress, ER stress, and dysbiosis in the gut are also the targets of BBR in the treatment of NAFLD. So, BBR improves NAFLD via targeting multiple pathophysiological procedures.

### 3.1. BBR Improves Insulin Resistance via Multiple Ways

**Figure 2:**
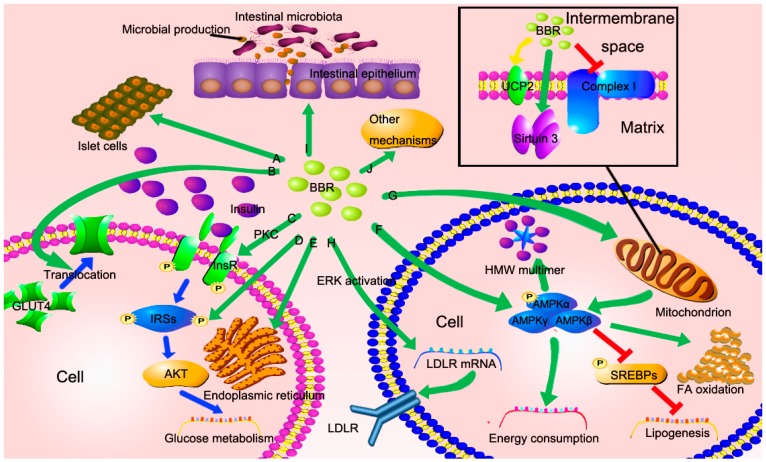
The potential mechanisms of berberine in the treatment of NAFLD. (**A**–**E**) BBR improves insulin resistance via directly triggering insulin secretion, increasing IRSs expression, increasing InsR expression through PKC activation, inducing GLUT4 translocation to cell membrane and decreasing ER stress; (**F**) BBR activates AMPK in both direct way and indirect way. The activation of AMPK decreases lipogenesis, increases energy consumption, promotes FA oxidation, and triggers adiponectin multimerization to HMW multimer; (**G**) BBR inhibits mitochondrial respiratory complex I and enhances AMP/ATP ratio, resulting in the activation of AMPK. In addition, BBR increases the expression of sirtuin 3, improving mitochondrial function and alleviating oxidative stress. It is controversial whether BBR induces UCP2 expression; (**H**) BBR stabilizes LDLR mRNA and increases LDLR expression, which depends on ERK activation; (**I**) BBR regulates intestinal microbiota, reduces the entry of microbial productions, and repairs gut permeability caused by damaged tight junctions; (**J**) Other mechanisms containing reduction of PCSK9 expression and DNA methylation may be involved.

There is a synergistic action between hyperlipoidemia and insulin resistance. When patients develop insulin resistance, the glucose-lowering effect of insulin weakens, while the lipogenesis effect of insulin still works, which results in the progression of NAFLD [31]. Increased evidence suggests that BBR can improve insulin resistance [32,33,34,35,36,37,38], and several potential mechanisms have been discovered (Figure 2A–E).

BBR directly induces insulin secretion in HIT-T15 cells, pancreatic islets, and normal mice [39]. Another route of BBR in the regulation of insulin resistance is via increasing glucose transporter 4 (GLUT4) translocation to the cell membrane in myotubes and decreasing lipid mass in adipocytes [32]. In addition, insulin signaling pathway can also be improved by BBR. Treatment with BBR increases expression of insulin receptor (InsR) mRNA and protein both in rat L6 cells and human hepatocytes (HepG2 and Bel-7402) in a dose- and time-dependent manner [36]. Further investigation indicates that the increased InsR gene expression is via activation of its promoter by protein kinase C (PKC) [36]. Insulin receptor substrate-2 (IRS-2) is another target of BBR. Treatment with BBR in rats upregulates IRS-2 messenger RNA (mRNA) and protein, which serves a significant role in the insulin signaling pathway [38]. BBR modifies Ser/Thr phosphorylation of IRS-1 and downstream Protein kinase B (PKB), also called AKT, leading to improved insulin signaling cascade [37]. Adiponectin, an adipokine secreted by adipocytes, is identified to increase insulin sensitivity [40]. Increased expression of adiponectin enhances insulin sensitivity via activation of AMPK [40,41]. Adiponectin possesses three multimers—trimer (LMW), hexamer (MMW), and high-molecular-weight (HMW) multimer—and the HMW adiponectin possesses more activity [42,43]. BBR reduces the adiponectin expression, but enhances adiponectin multimerization to HMW multimer via activation of AMPK, thus leading to enhanced insulin sensitivity [44].

The anti-inflammatory potential and anti-ER stress effect of BBR may be involved in reversing insulin resistance. Interleukin-6 (IL-6) and tumor necrosis factor-𝛼 (TNF-𝛼) induced by palmitate, which lead to inflammation and insulin resistance, are effectively diminished by BBR in a dose-dependent manner [37]. Further, BBR reduces cyclooxygenase-2 (COX-2) protein, decreases phosphorylation state of c-Jun N-terminal kinase 1 (JNK1) and reduces the mRNA levels of proinflammatory cytokines, resulting in an anti-inflammation effect [45,46]. Because inflammation also plays a critical role in the pathogenesis of NAFLD, the anti-inflammatory effect of BBR can alleviate the condition of NAFLD directly. Study showed that the expression of oxygen-regulated protein 150 (ORP150) was reduced when pretreated with BBR in HepG2 cells, while the phosphorylation of PKR-like eukaryotic initiation factor 2α kinase (PERK) and eukaryotic translational initiation factor 2α (eIF2α) was blocked accompanied by increased phosphorylation of IRS-1 Tyr and AKT Ser473 [47]. ORP150, PERK and eIF2α are considered as the molecular markers of ER stress [47]. A more recent study suggested that the beneficial effect of BBR on ER stress induced lipogenesis was partly via the activating transcription factor 6 (ATF6)/sterol regulatory element-binding protein-1c (SREBP-1c) pathway in vitro [48].

Because of the synergistic effect between NAFLD and insulin resistance, improvement of fat metabolism reverts insulin resistance to some extent. Peroxisome proliferator–activated receptors (PPARs) regulate fat metabolism and energy homeostasis involving β-oxidation and adipogenic processes [35,49]. Study indicated that BBR reduced PPARγ and fatty acid translocase (FAT) protein expressions, and decreased fatty acid uptake, thus leading to improved glucose uptake in myotubes [35]. Moreover, the expression of PPARγ2, C/EBPα, adiponectin, and leptin mRNA was downregulated by BBR in human preadipocytes and metabolic syndrome patients, suggesting that improved fat storage and adjusted adipokine profile play a role in restoring insulin resistance [50].

### 3.2. BBR Reduced Lipid Accumulation via Regulating AMPK Phosphorylation

AMPK is a kind of highly conservative, heterotrimeric, serine threonine protein kinase, which consists of a catalytic subunit, AMPKα, and two regulatory subunits, AMPKβ and AMPKγ [51,52]. AMPK is considered as a pivotal role in systemic energy metabolism [51,53]. Study showed that BBR acutely activated AMPK in both L6 myotubes and 3T3-L1 adipocytes [32]. Both central and peripheral administration of BBR induce AMPK activity [54]. Increased evidence indicates that AMPK was activated by BBR via increasing phosphorylation of AMPK [32,54,55]. The AMPK activity induced by BBR does not depend on the activity of either liver kinase B1 (LKB1) or calmodulin-mediated kinase kinase β (CaMKKβ) [56]. LKB1 and CaMKKβ are the main kinases that activate AMPK via phosphorylation of Thr172 [57,58,59]. However, it is controversial whether BBR activates AMPK in a direct way. Evidence indicates that treatment with BBR inhibits mitochondrial respiratory complex I and increases AMP/ATP ratio, thus leading to the activation of AMPK [56,60]. So, it is possible that there is both a direct and indirect way contribute to the activation of AMPK by BBR (Figure 2F).

The activation of AMPK downregulates lipogenesis associated genes, and upregulates energy consumption, resulting in reduced lipid accumulation and improved liver condition. The phosphorylation of AMPK directly suppresses activity of SREBP-1c and -2 via Ser372 phosphorylation and blockades SREBP-1c cleavage and nuclear translocation, leading to decreased SREBP-1c target gene, involving acetyl-CoA carboxylase 1 (ACC1), fatty acid synthase (FAS), and stearoyl CoA desaturase 1 (SCD1) transcription and translation [33]. In addition, the mRNAs of two key enzymes of cholesterol biosynthesis, 3′-hydroxylmethyl glutaryl coenzyme A synthetase (HMGCS) and 3’-hydroxylmethyl glutaryl coenzyme A reductase (HMGCR), are also reduced by activation of AMPK, which is consistent with decreased SREBP-2 [33]. Therefore, reduced expression of ACC1, FAS, SCD1, HMGCS, and HMGCR leads to decreased triglyceride and cholesterol biosynthesis, thus improving hepatic steatosis [33]. Furthermore, BBR metabolite, columbamine, also presents the potential effects on reducing TG level, which is also via activation of AMPK [61].

### 3.3. BBR Improves Mitochondrial Function and Alleviates Oxidative Stress

As mentioned above, BBR can block mitochondrial respiratory complex I. However, recent study demonstrated that BBR reduced mitochondrial reactive oxygen species (ROS) generation and improved hepatic mitochondrial dysfunction in obese rats probably via activating the mitochondrial sirtuin (SIRT3) [62]. Furthermore, BBR ameliorates mitochondrial dysfunction in skeletal muscle partially via promoting mitochondrial biogenesis [63]. Further study showed that the function of BBR on mitochondrion was inhibited in SIRT1 knocked-down cells, which suggested that SIRT1 may regulate the effect of BBR on mitochondrion [63]. It seems that BBR has a paradoxical effect on mitochondrion. Interestingly, living cells grow normally even in the absence of complex I activity in vitro [60], which may partly account for the paradoxical effect of BBR on mitochondrion. Nevertheless, the reason why cells grow normally in the absence of complex I activity is unclear, which should be further explored.

Uncoupling protein-2 (UCP2), a mitochondrial inner membrane carrier protein, is expressed at a low level in normal hepatocytes and at a high level in Kupffer cells [64]. Like UCP1, UCP2 is associated with heat production by inhibiting mitochondrial respiration via mediating proton leaking, thus reducing ATP synthesis. Study shows that UCP2 is associated with fat accumulation, oxidative stress, insulin resistance, and increased serum fatty acid level [65,66,67]. Increased oxidative stress and ROS induced the expression of UPC2 in mice with NAFLD [67]. UCP2 expression is increased in high-fat diet-fed rats and administration of BBR reduces the expression of UCP2 mRNA and protein [68]. However, further evidence suggests that upregulation of UCP2 in hepatocytes also improves the condition of NAFLD. The UCP2-866 A/A genotype in human induces hepatic UCP2 expression, but decreases the risk of nonalcoholic steatohepatitis [69]. So, why do both upregulation and downregulation of UCP2 expression lead to prevention of NAFLD? Is UCP2 really required for the effect of BBR on NAFLD (Figure 2G)?

### 3.4. BBR Reduces Serum Cholesterol via A Distinctive Mechanism

Distinct from statins, the cholesterol-lowering effect of BBR is through stabilization of low-density lipoprotein receptor (LDLR) mRNA independent of SREBP [70]. The prolonged existence of LDLR mRNA increases the expression of LDLR, thus leading to increased cholesterol catabolism. Additional evidence elucidates that the effect of stabilization of LDLR mRNA induced by BBR is dependent on extracellular signal-regulated kinase (ERK) activation (Figure 2H) [70].

### 3.5. The New Role of BBR in Gut Microenvironment

It is well known that BBR possesses antimicrobial activity, which is used to treat diarrhea and gut infections. Evidence indicates that the regulation of BBR on gut microenvironment may partially account for the improved NAFLD condition (Figure 2I). Treatment of BBR regulates intestinal microbiota, reduces fat absorbing, and decreases inflammation through reducing the entry of exogenous antigens and enhancing short-chain fatty acid in the gut [71,72]. Besides, impaired gut permeability is repaired by BBR via reverting damaged tight junctions in intestinal epithelium [73,74]. However, the mechanism underlying these phenomena is not completely known.

### 3.6. Other Potential Mechanisms

*PCSK9* (encoding proprotein convertase subtilisin/kexin type 9) was discovered as a third locus associated with autosomal dominant hypercholesterolemia in 2003 [75]. Neural apoptosis regulated convertase-1 (NARC-1), encoded by *PCSK9*, is highly expressed in the liver and associated with cholesterol homeostasis [75]. Mutant of *PCSK9* is associated with lifelong reduced serum LDL cholesterol (LDL-c), leading to decreased risk of cardiovascular events [76]. Evidence showed that PCSK9 expression was decreased in high-fat diet and BBR-fed mice, suggesting PCSK9 may be a new molecular target of BBR [77]. In addition, the metabolic profiles containing sphingomyelin (SM), phosphatidylcholine (PC), lysophosphatidylcholine (LysoPC), 13-hydroperoxy-9, 11-octadecadienoic acid (13-HpODE), eicosatrienoic acid, docosatrienoic acid, eicosenoic acid, glucose, maltose, and cholesterol change after BBR treatment [78,79].

As the development of bioinformatics, new methods and technologies enable us to gain insight into the genetic mechanism of BBR in the treatment of NAFLD, uncovering another new aspect of BBR function. Recent study conducted by our department using the methods of bioinformatics showed that the expression of 881 mRNAs and 538 long noncoding RNAs (lncRNAs) was restored by treatment of BBR in the steatotic liver [80]. Among them, a significant association was found between a conserved lncRNA, *MRAK052686*, and antioxidant factor nuclear respiratory factor 2 (Nrf2) [80]. Treatment of BBR increased both *MRAK052686* and Nrf2 expression [80]. These findings indicate that BBR improves NAFLD via a global modulation of hepatic mRNA and lncRNA expression profiles [80].

Additionally, DNA demethylation is also involved in the mechanism of BBR. We demonstrated that the methylation of microsomal triglyceride transfer protein (MTTP) promoter was decreased and the expression of MTTP is increased by BBR in rats [81]. MTTP locates in ER and is associated with VLDL assembly. Then, increased expression of MTTP promoted VLDL assembly and secretion, thus leading to improved NAFLD [81]. However, the precise mechanism of it is not conclusively determined (Figure 2J).

## 4. Future Perspective

Although no drug is approved in pharmacological therapy for NAFLD, the promising effect of BBR manifested both in animal experiments and clinical trials makes it a candidate for future pharmacological therapy against NAFLD. Lifestyle modification such as weight loss through calorie restriction and exercise does have beneficial effects on NAFLD. However, the mechanisms behind it are not completely uncovered. Both lifestyle modification and berberine can improve insulin resistance, regulate intestinal microbiota, upregulate energy consumption, and reduce serum cholesterol level, but they may work via different pathways. For instance, physical activity increases energy consumption mainly via increasing the demand of energy, while berberine increases energy consumption mainly via promoting fatty acid oxidation. Dietary intervention improves NAFLD via reducing the input of TGs, which is different from berberine. Berberine improves NAFLD via inhibiting lipogenesis, which is different from dietary intervention. Lifestyle modifications improve the condition of NAFLD, but are limited by sustainability due to patient motivation and difficulty with adherence. Lifestyle modification alone is not enough to revert NAFLD in serious conditions. In order to facilitate the process of BBR clinical application, there are still a number of issues to be solved: (a) The mechanisms of BBR in the treatment of NAFLD and the pathogenesis of NAFLD should be further investigated, especially in immunology and the gut microenvironment. Recent study suggested that dysfunction of lipid metabolism regulation in NAFLD promoted hepatocarcinogenesis via inducing a selective loss of CD4^+^ T lymphocytes in liver and impairing anti-tumor surveillance [82]. However, few studies have conducted research about the association between effect of BBR on NAFLD and immunity so far; (b) To increase the effect and reduce the side effect of BBR, although evidence indicates that BBR induced adverse effects are mild and tolerant [27,28,29,83], development of appropriate berberine derivative may be required. To date, some berberine derivatives have already been investigated [84,85]; (c) Drug combination is another alternative strategy to enhance the effect of BBR. Combination of BBR and plant stanols shows a synergistic effect on deceasing serum cholesterol in rats [86]. However, be well aware of the drug interactions; (d) Other factors containing route of administration and drug dose should be considered.
